# Exercise-Induced Rhabdomyolysis and Stress-Induced Malignant Hyperthermia Events, Association with Malignant Hyperthermia Susceptibility, and *RYR1* Gene Sequence Variations

**DOI:** 10.1155/2013/531465

**Published:** 2013-02-10

**Authors:** Antonella Carsana

**Affiliations:** ^1^Department of Molecular Medicine and Medical Biotechnology, University of Naples Federico II, 80131 Naples, Italy; ^2^CEINGE-Biotecnologie Avanzate, 80145 Naples, Italy

## Abstract

Exertional rhabdomyolysis (ER) and stress-induced malignant hyperthermia (MH) events are syndromes that primarily afflict military recruits in basic training and athletes. Events similar to those occurring in ER and in stress-induced MH events are triggered after exposure to anesthetic agents in MH-susceptible (MHS) patients. MH is an autosomal dominant hypermetabolic condition that occurs in genetically predisposed subjects during general anesthesia, induced by commonly used volatile anesthetics and/or the neuromuscular blocking agent succinylcholine. Triggering agents cause an altered intracellular calcium regulation. Mutations in *RYR1* gene have been found in about 70% of MH families. The *RYR1* gene encodes the skeletal muscle calcium release channel of the sarcoplasmic reticulum, commonly known as ryanodine receptor type 1 (RYR1). The present work reviews the documented cases of ER or of stress-induced MH events in which *RYR1* sequence variations, associated or possibly associated to MHS status, have been identified.

## 1. Introduction

**Figure 1:**
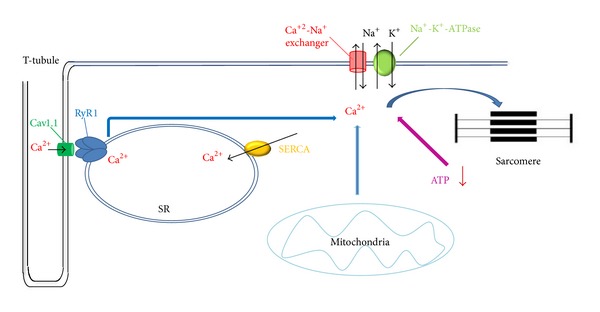
Schematic representation of a skeletal muscle cell and of Ca^2+^ and Na^+^ ion fluxes across the sarcolemma and sarcoplasmic reticulum (SR). Activation of Cav1.1 by membrane depolarization causes the RyR1 channel to open and to release Ca^2+^ from SR, thus triggering muscle contraction. Ca^2+^ concentration is regulated by the Ca^2+^-ATPase membrane pump (SERCA) that sequesters Ca^2+^ in the SR and by the Na^+^-K^+^-ATPase membrane pump and the Ca^2+^-Na^+^ antiport that exchange Ca^2+^ for Na^+^ across the sarcolemma. Regulation of calcium flux may be disrupted at any of these sites. ATP depletion, by consumption during muscle contraction, or reduced ATP production, results in intracellular Ca^2+^ increasing, muscle contraction, and continued energy consumption, leading to rhabdomyolysis.

Rhabdomyolysis is an acute syndrome determined by a direct or indirect muscle injury. It results from skeletal muscle breakdown and massive release of the intracellular content into blood circulation, which can lead to potentially fatal events, such as acute renal failure, hyperkalemia, and other metabolic complications [1, 2]. The etiology of rhabdomyolysis is broad and includes inherited diseases, drugs, toxins, muscle compression, overexertion, and infections. Regardless of the mechanism, these muscle injuries ultimately lead to a leakage of Ca^2+^ ions into the intracellular space, and the excess of Ca^2+^ ions gives rise to a persistent muscle contraction that ends in energy depletion and cell death (Figure 1) [1]. Rhabdomyolysis syndrome may also occur as a result of a strenuous or not strenuous physical exercise (exertional rhabdomyolysis or ER) often in hot and humid climates. Although anyone may develop ER under extreme physical and environmental conditions, some individuals seem to be more predisposed than others, suggesting a genetic link. The most commonly identified predisposing conditions of ER are deficiencies of carnitine palmitoyltransferase II (*CPT2* gene, OMIM *600650), myophosphorylase (McArdle disease, *PYGM* gene, OMIM *608455), and myoadenylate deaminase (*AMPD1* gene, OMIM +102770). Events similar to those occurring in ER are triggered after exposure to anesthetic agents in malignant hyperthermia susceptible (MHS) patients. Therefore, an association between ER and malignant hyperthermia (MH) has been investigated and reported [3–10]. However, two studies on the effect of exercise on thermoregulatory and metabolic responses in MHS subjects gave controversial results [11, 12]. Moreover, cases of MH-like events in the absence of anesthetic agents, and caused by high environmental or core body temperature, or even by emotional stress, have been reported [13–16].

Malignant hyperthermia (OMIM #145600) is an autosomal dominant hypermetabolic condition that occurs in genetically predisposed subjects during general anesthesia, induced by commonly used volatile anesthetics and/or the neuromuscular blocking agent succinylcholine. Triggering agents cause an altered intracellular calcium regulation. An MH attack, unless immediately recognized and treated, is often fatal. Clinical symptoms of a classic MH attack are accelerated muscle metabolism, muscle contractions, metabolic acidosis, tachycardia, and hyperthermia. These symptoms are correlated with some altered biochemical parameters, such as metabolic acidosis with increased pCO_2_ and lactate production and release of potassium and muscle proteins, as creatine kinase and myoglobin, into the blood. Frequent late events are damage of kidney function due to massive myoglobin release and/or a diffuse intravascular coagulation, which is often the main cause of death [17]. The prevalence of MH episodes is estimated to range from 1 : 10,000 to 1 : 220,000 [17]. Malignant hyperthermia susceptibility can be diagnosed by an *in vitro* test, based on the differential contractile response of normal (MHN) and MHS muscles to caffeine and halothane. Protocols for MH contracture testing of human skeletal muscle have been developed by the European [18] and North American [19] MH Groups, namely, *in vitro* contracture test (IVCT) and caffeine halothane contracture test (CHCT), respectively. A considerable genetic heterogeneity has been reported for MH. Six genetic *loci* (MHS1, OMIM #180901; MHS2, OMIM #154275; MHS3, OMIM #154276; MHS4, OMIM #600467; MHS5, OMIM #601887; MHS6, OMIM #601888-6), associated with MH, have been identified. About 70% of affected families are linked to the MHS1 *locus*, where the *RYR1* gene encoding the skeletal muscle calcium release channel of the sarcoplasmic reticulum, commonly known as ryanodine receptor type 1 (RyR1), maps. Dantrolene is an RyR1 antagonist that blocks calcium release from the sarcoplasmic reticulum stores and is the only specific agent available for the treatment of an MH attack. Less than 1% of MHS cases can be attributed to mutations in the *CACNA1S* gene (*locus* MHS5) encoding the *α*1S subunit of the voltage-dependent L-type calcium channel of the skeletal muscle, Cav1.1. Only three MH-causing mutations identified in the *CACNA1S* gene were hitherto functionally characterized [20–22]. RyR1 and Cav1.1 are the two major proteins involved in the excitation-contraction coupling in skeletal muscle.

The aim of this paper is to review the documented cases of ER or of stress-induced MH events in which sequence variations (SVs) of the *RYR1* gene, associated or possibly associated to MHS, have been identified.

## 2. Methods

The PubMed and Web of Science databases were consulted to search for studies on documented cases of ER or of stress-induced MH events in which *RYR1* SVs, associated or possibly associated to MHS, have been identified. Search terms included “*RYR1*,” “mutation,” “malignant hyperthermia,” “exercise,” “heat stress,” “stress-induced malignant hyperthermia,” and “nonanesthetic malignant hyperthermia.” Single-nucleotide polymorphism (SNP) databases (http://www.ncbi.nlm.nih.gov/snp, http://www.dmd.nl/nmdb2/variants.php?select_db=RYR1) were also searched. Three different programs, namely, PMut (http://mmb.pcb.ub.es/PMut/), SIFT (http://sift.jcvi.org/), and PolyPhen-2 (http://genetics.bwh.harvard.edu/pph2/), were used to predict the pathological character of *RYR1* SVs which have not been functionally characterized. PMut is based on the use of neural networks trained with a very large database of human disease-associated mutations and neutral SVs [23] and combines sequence alignment/position-specific scoring matrix with structural factors; score >0.5 predicts a pathological effect. SIFT is based on the degree of conservation of amino acid residues in sequence alignments derived from closely related sequences [24]. The SIFT scores range from 0 to 1; the amino acid substitution is predicted as damaging if the score is ≤0.05 and as tolerated if the score is >0.05. PolyPhen-2 predicts the effects of an amino acid substitution using both structure and sequence information [25] and classifies variants as “probably damaging,” “possibly damaging,” or “benign,” based on pairs of false positive rate thresholds.

## 3. Results

### 3.1. *RYR1* Gene Sequence Variations (SVs) in ER and Stress-Induced MH Patients

Thus far, more than 300 missense SVs have been identified in the *RYR1* gene (http://www.ncbi.nlm.nih.gov/snp, http://www.dmd.nl/nmdb2/variants.php?select_db=RYR1). Some *RYR1* SVs have been characterized by *in vitro* functional studies. The demonstration that a SV alters the kinetic properties of the RyR1 channel allows to define its role in the pathogenesis of MHS. Various methods have been developed to characterize the function of RyR1 variants: analysis of calcium release in human primary myotubes [26–28] and in immortalized B lymphocytes from patients or after expression by transfection in various cell types [29–31], determination of the channel openings in a ryanodine binding assay [32], and a metabolic test *in vitro* based on the measurements of proton release rate in immortalized B lymphocytes from patients [33]. MHS-associated *RYR1* mutations cause the channels to become hypersensitive to activation by electrical and pharmacological (caffeine, halothane, 4-chloro-m-cresol) stimuli. Identification of causative *RYR1* mutations is an aid to the diagnosis of MHS. In fact, although the IVCT/CHCT are the gold standard to establish the risk of MHS, an individual harboring an MH causative mutation can be considered MHS even without an IVCT/CHCT result (http://www.emhg.org). Furthermore, genetic analysis is crucial to identify and evaluate the few cases of discordance between genotype, characterized by the presence of a causative mutation, and MHN-typed phenotype [34, 35]. A retrospective study reported these discordant cases in approximately 2.6% of *RYR1 *mutation-positive families [35]. Such discordant subjects are regarded as MHS for clinical purposes on the basis of genetic data alone, since they bear a causative mutation [34, 35].

Table 1 shows a list of *RYR1* gene missense SVs and the corresponding amino acid substitutions, identified in patients who experienced ER or stress-induced MH events [10, 13–16, 36–38]. Four *RYR1* SVs, corresponding to the amino acid substitutions p.R163C, p.G341R, p.G2434R, and p.T4826I, have already been demonstrated to be causative of MHS (http://www.emhg.org). The p.R3983C substitution was identified in two unrelated children who had fatal, nonanesthetic awake episodes associated with febrile illness and heat stress [15]. One of the children also had the variant p.D4505H. Interestingly, the child who only had the p.R3983 variant also had an MH attack during general anesthesia with halothane. These two SVs were functionally characterized by evaluating the caffeine sensitivity of Ca^2+^ release in transfected myotubes. Both p.R3983C and p.D4505H RyR1 channel variants exhibit an increase in the sensitivity to activation by caffeine, although the effect of the p.R3983C substitution alone is quite modest [15]. The SVs p.R401C, p.A933T, p.G2160S, p.R2336H, p.T4288_A4290dup, p.T4294 M, p.L4320_R4322dup, and p.R4645Q were reported to be absent in at least 100 control chromosomes. Instead, the p.S1342G and the p.S1352G variants are present among the African American population with a frequency of 4% and 2.7%, respectively [39], indicating that they are neutral polymorphic changes in RyR1. The p.R2336H, p.T4288_A4290dup, p.L4320_R4322dup, and p.R4645Q SVs have already been reported in MHS families [40–42].

### 3.2. In Silico Analysis of *RYR1* Variants Reported in Patients Who Experienced ER and Stress-Induced MH Events

To predict the pathological character of p.E209 K, p.R401C, p.A933T, p.G2160S, p.R2336H, p.T4294 M, and p.R4645Q SVs, I tested them with 3 different prediction programs, namely, PMut (http://mmb.pcb.ub.es/PMut/) [23], SIFT (http://sift.jcvi.org/) [24], and PolyPhen-2 (http://genetics.bwh.harvard.edu/pph2/) [25].  Table 2 shows the results obtained by this analysis. The p.R401C, p.A933T, and p.R2336H variants were predicted to have a pathological character, while the predictions generated for p.E209 K, p.G2160S, p.T4294 M, and p.R4645Q variants were divergent. The p.E209 K variant, that has been predicted to be neutral by two programs and only possibly damaging by PolyPhen-2, has been found in association with p.R2336H in one patients who experienced stress-induced MH events and was typed MHS by CHCT (see Table 1) [36]. All the programs tested predict a pathological effect for the p.R2336H variant, that could be the molecular basis of both phenotypes. However, functional studies are needed to conclusively define the exact pathogenic effects of this amino acid substitution and to assess if it is the cause of stress-induced MH events in the patient. 

Wappler et al. [10] found causative mutations (p.R163C, p.G341R, and p.G2434R) in only three out of ten MHS patients who experienced ER. They screened only eight *RYR1* exons located in the hotspot region; therefore, this limited analysis can explain the low mutation detection rate. Moreover, Sambuughin et al. [39], by sequencing the *RYR1* cDNA, found putative causative SVs (p.A933T and p.T4294 M) in only two out of six ER/MHS patients studied. In the remaining cases, the ER/MHS phenotype could be caused by *RYR1* SVs which may escape the *RYR1* cDNA screening because they determine unbalanced allelic expression [43–46] or, alternatively, could be caused by mutations in other candidate MHS *loci* genes. 

## 4. Conclusions and Perspectives

ER and stress-induced MH events are syndromes with diverse etiologies that afflict particularly military recruits in basic training and athletes. This paper reports an overview of the literature on cases associated with MHS and with* RYR1* causative mutations or putative causative SVs. The possible disease-causing role of SVs, identified in patients who experienced ER and stress-induced MH events and that have not been functionally characterized, was investigated by computational analysis by using three different approaches, to increase the predictive power. Although only the molecular characterization of RyR1 channel variants can define the functional impact of a given SV, in silico predictions, which are fast and relatively inexpensive methods, may filter out SVs that are unlikely to affect protein function and allow phenotype prediction based on the biochemical severity of the amino acid substitution and on the protein sequence and structural information. Overall, the data presented in this paper emphasize the concept that some *RYR1* SVs are associated with both phenotypes and underline the importance of performing contracture testing and *RYR1* variant screening in these patients.

A mouse model of heat- and anesthetic-induced MHS has been created by introducing the p.Y522S mutation in the *RYR1* gene [47]. Only mice which are heterozygous for the p.Y522S mutation (RyR1^Y522S/wt^) are viable and exhibit whole body contractions and elevated core temperatures in response to anesthetic exposure or heat stress [47]. Elevated environmental temperatures induce muscle contractures, rhabdomyolysis, and death in these mice. The Ca^2+^ leaking caused by the p.Y522S mutation, combined with temperature, generates increases in reactive nitrogen species and S-nitrosylation of the mutant channel that enhances RyR1 channel activity. Ultimately, the exposure to elevated temperatures produces abnormal muscle contractures in the RyR1^Y522S/wt^ mice [48]. Recently, it has been reported that AICAR, an activator of the AMP-activated protein kinase (AMPK), prevents Ca^2+^ leaking, generation of reactive oxygen and nitrogen species, and heat-induced sudden death in RyR1^Y522S/wt^ mice [49]. The effect of AICAR is not due to an increase in AMPK activity but to the inhibition of RyR1 channel activity. On the basis of these results, Lanner et al. [49] proposed “the potential use of AICAR for prophylactic treatment in humans with enhanced susceptibility to exercise and/or heat-induced sudden death associated with RyR1 disease mutations.” Moreover, studies on the effects of prior eccentric exercise on isolated mouse RyR1^Y522S/wt^ muscle indicated that high-force eccentric contractions, run under nonthermally stressful conditions, may attenuate the thermal stress-induced loss of function [50]. This finding can have important implications because it suggests that the exercise-induced muscle injury may mitigate the severity of stress-induced MH episodes, possibly in humans as well.

## Figures and Tables

**Table 1:** *RYR1* sequence variants reported in patients who experienced ER and stress-induced MH events.

Nucleotide change	Exons	Aminoacid change	MH-causative mutation (http://www.emhg.org)	Unrelated patients (*n*)	Regions of the *RYR1* gene investigated	dbSNP	MH status	References
c.487C>T	6	R163C	Yes	1 1	gDNA hot spot	rs118192161	MHS n.d	[10] [13]
c.625G>A c.7007G>A	7 43	E209K/ R2336H		1	cDNA complete	— rs112563513	MHS	[36]
c.1021G>A	11	G341R	Yes	1	gDNA hot spot	rs121918592	MHS	[10]
c.1201C>T	12	R401C		2	cDNA hot spot	—	MHS	[16]
c.2797G>A c.4024A>G c.4055C>G	23 28 28	A933T/ S1342G/ A1352G		1	cDNA complete	rs148623597 rs34694816 rs112105381	MHS	[39]
c.4024A>G	28	S1342G		3	cDNA complete	rs34694816	MHS	[37, 39]
c.4024A>G c.4055C>G c.12861_12869dup c.12881C>T	28 28 91 91	S1342G/ A1352G/ T4288_A4290dup/ T4294M		1	cDNA complete	rs34694816 rs112105381 — —	MHS	[39]
c.2797G>A c.6478G>A	28 39	S1342G/ G2160S		1	cDNA complete	rs34694816 rs143398211	MHS	[39]
c.7300G>A	45	G2434R	Yes	1	gDNA hot spot	rs121918593	MHS	[10]
c.11947C>T	87	R3983C	Yes	1*	gDNA (106 exons)	—	n.d.	[15]
c.11947C>T c.13513G>C	87 92	R3983C/ D4505H	Yes Yes	1*	gDNA (106 exons)	—	MHS	[15]
c.12959_12967dup c.13934G>A	91 95	L4320_R4322dup/ R4645Q		1*	gDNA (106 exons)	—	n.d.	[14]
c.14473C>T	100	T4826I	Yes	1*	cDNA complete	rs121918595	n.d.	[38]

*patients who experienced stress-induced MH events; n.d.: not determined. Nucleotide substitutions were numbered on the cDNA sequence (GenBank NM_000540.2); gDNA: genomic DNA.

**Table 2:** In silico analysis of *RYR1* sequence variants reported in patients who experienced ER and stress-induced MH events.

Sequence variant	PMut	SIFT	Polyphen-2
p.E209K	**0.6598**	0.29	Possibly damaging
**p.R401C**	**0.8400**	**0.04**	**Probably damaging**
**p.A933T**	**0.5969**	**0.01**	**Probably damaging**
p.G2160S	0.2159	0.49	Possibly damaging
**p.R2336H**	**0.8377**	**0.00**	**Probably damaging**
p.T4294M	**0.8994**	0.11	Benign
p.R4645Q	**0.8261**	**0.00**	Benign

Scores predicting pathological effect are in bold: PMut, > 0.5; SIFT ≤ 0.05. Polyphen-2 classifies the sequence variants as probably damaging, possibly damaging, or benign.
